# Supplementary material for: Muscleblind-like proteins are novel modulators of the tumor-immune microenvironment
Source: PLoS One. 2025 Apr 30;20(4):e0321148. doi: 10.1371/journal.pone.0321148 (PMC12043120; doi:10.1371/journal.pone.0321148)
Supplement: S1 File — (PDF) [file pone.0321148.s001.pdf]

Supplementary Figure 1

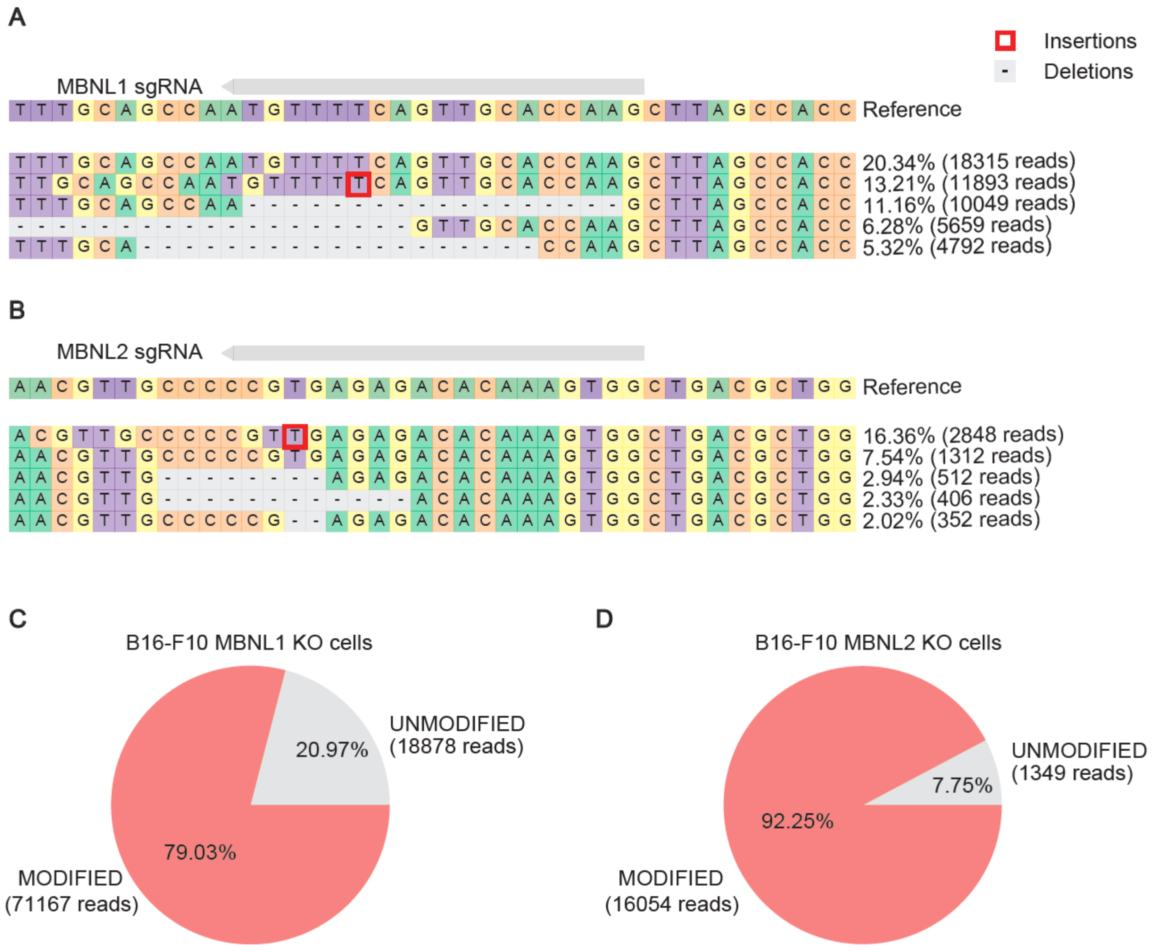

Figure S1: Related to Figure 2.
(**A**) 5 most abundant alleles generated following treatment of Cas9 expressing B16-F10 cells with a gRNA targeting Mbnl1.
(**B**) 5 most abundant alleles generated following treatment of Cas9 expressing B16-F10 cells with a gRNA targeting Mbnl2.
(**C**) Pie chart of the fraction of alleles isolated that contain an indel of any kind in Cas9 expressing B16-F10 cells with a gRNA targeting Mbnl1.
(**D**) Pie chart of the fraction of alleles isolated that contain an indel of any kind in Cas9 expressing B16-F10 cells with a gRNA targeting Mbnl2.

Supplementary Figure 2

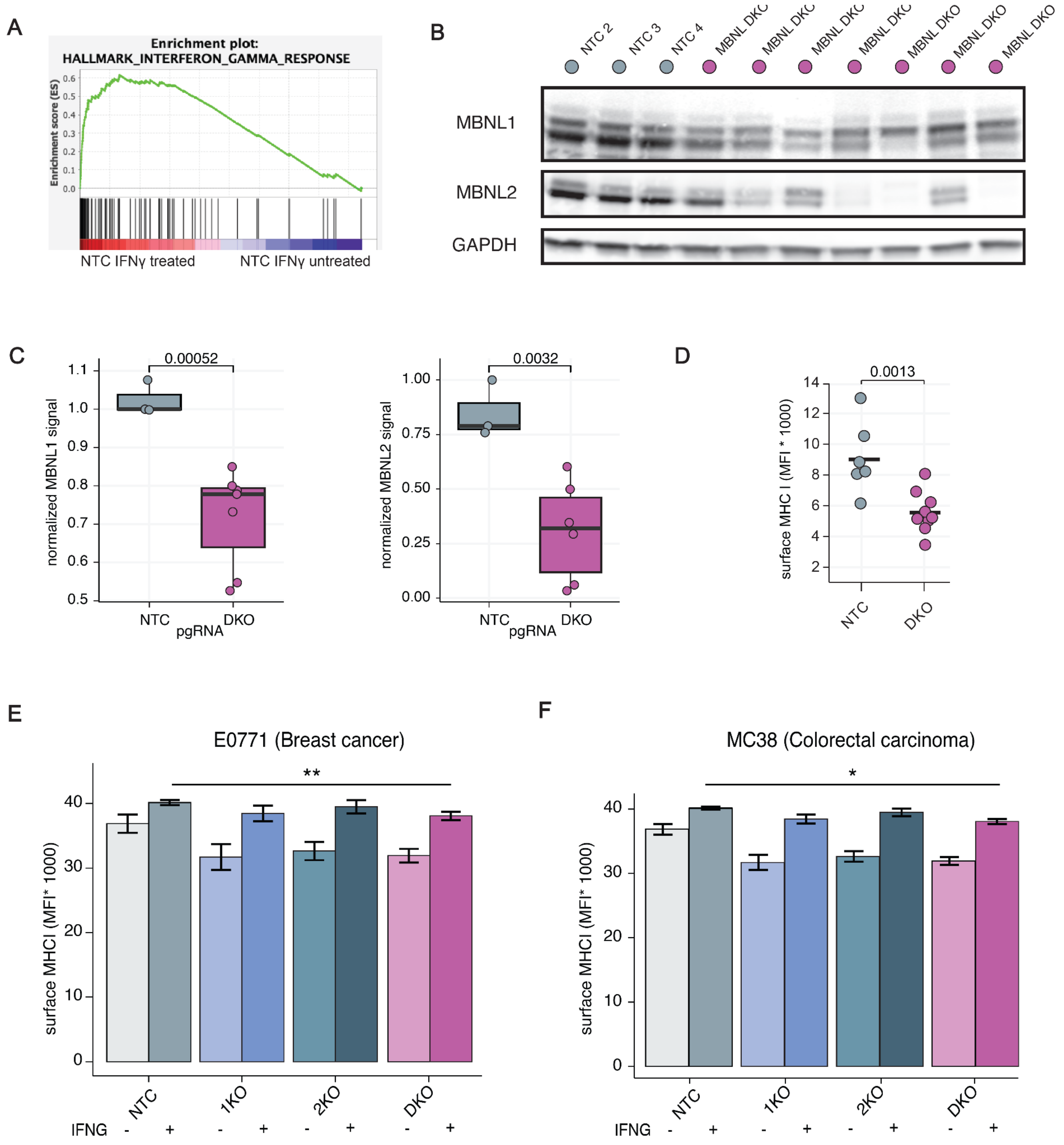

Figure S2: Related to Figure 2.

(**A**) Gene set enrichment of Hallmark Interferon Gamma Response in IFNγ treated vs untreated non-targeting control (NTC) B16-F10 cells.

(**B**) Western blot analysis of normalized MBNL1 expression in monoclonal B16-F10 cells isolated from either polyclonal Cas9-expressing B16-F10 cells treated with either an NTC or MBNL DKO pgRNA. P value is from a two-sided Student's t-test.

(**C**) Western blot analysis of MBNL2 expression, normalized to GAPDH signal, in monoclonal B16-F10 cells isolated from either polyclonal Cas9-expressing B16-F10 cells treated with either an NTC or MBNL DKO pgRNA. P value is from a two-sided Student's t-test.

(**D**) Surface MHC Class I expression in NTC and MBNL DKO cells.
(**E-F**) Surface MHC Class I expression across MBNL genotypes with and without IFNγ stimulation for E0771 breast cancer and MC38 colorectal carcinoma cell lines, respectively. (1KO = *Mbnl1* knockout, 2KO = *Mbnl2* knockout).

Supplementary Figure 3

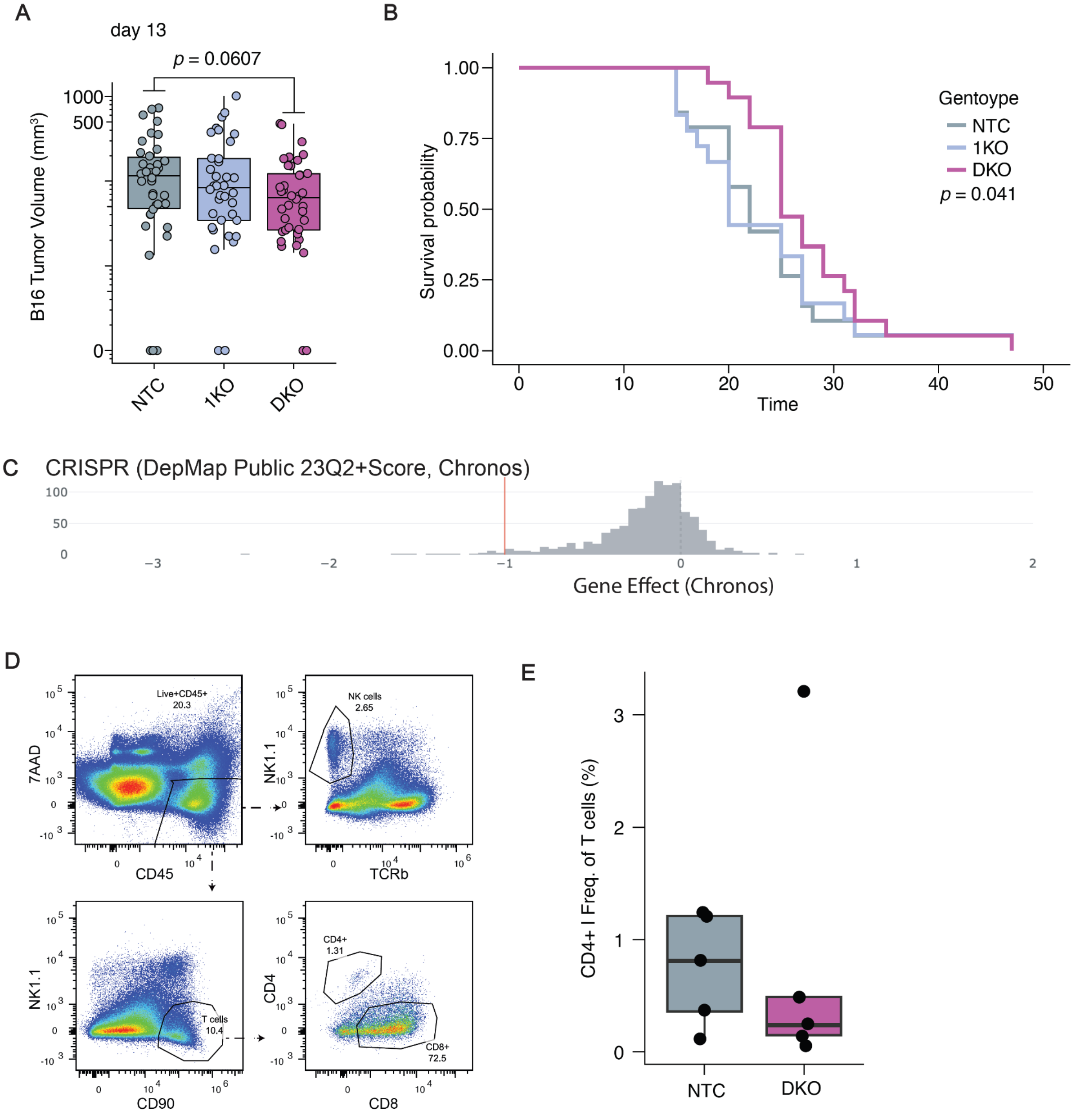

Figure S3: Related to Figure 3.

(**A**) B16-F10 *in vivo* tumor volume (mm^3) for non-targeting control (NTC), *Mbnl1* single knockout and *Mbnl1-2* double knockout. *P* value from a two-sided Wilcoxon signed-rank test.

(**B**) Kaplan-Meier survival curve for mice with NTC, 1KO and DKO B16-F10 tumors. P value from a log-rank test.

(**C**) DepMap chronos score for the CRISPR perturbation of MBNL1 across cancer cell lines.

(**D**) Gating strategy for analysis of B16-F10 tumor immune infiltrates.

(**E**) Frequence of CD4+ T cells as a percentage of total T cells in NTC and DKO B16-F10 tumors.

Supplementary Figure 4

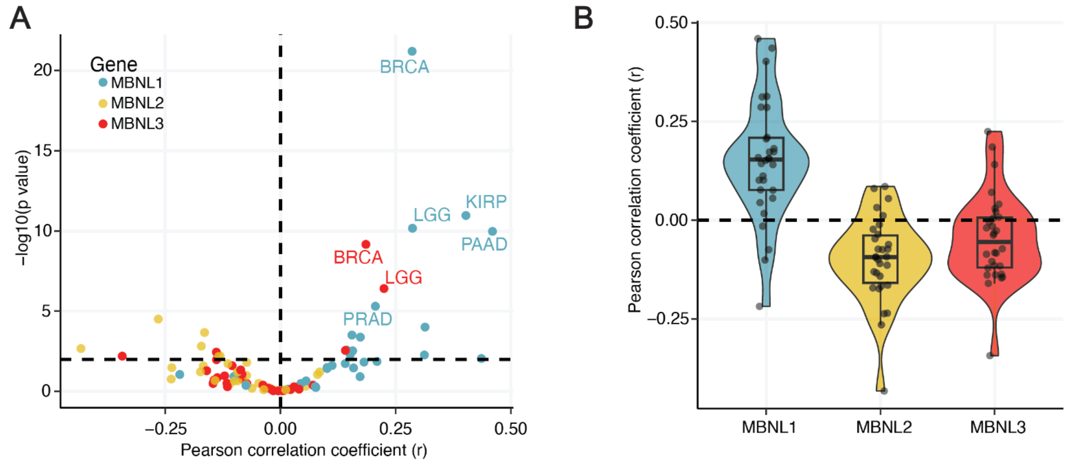

Figure S4: Related to Figure 4.
(**A-B**) Volcano (A) and violin (B) plots of Pearson correlation coefficients for MBNL1-3 expression with cytolytic T cell (CYT) score (geometric mean of *PRF1* and *GZMB* expression) (20) across TCGA cancer datasets.
