# Supplementary material for: Muscleblind-like proteins are novel modulators of the tumor-immune microenvironment
Source: PLoS One. 2025 Apr 30;20(4):e0321148. doi: 10.1371/journal.pone.0321148 (PMC12043120; doi:10.1371/journal.pone.0321148)

Uncropped gels

- All gels imaged with Li-cor Odyssey near-infrared scanner

<u>Figure 2, panel A</u>

Three side by side gels, all technical replicates of same samples

Gel stained with anti-MBNL1

Lanes (left to right, same in all 3 gel images below)

1	2	3	4	5
Ladder	B16-F10 Cas9 WT	B16-F10 Cas9 MBNL1 KO	B16-F10 Cas9 MBNL2 KO	B16-F10 Cas9 MBNL DKO

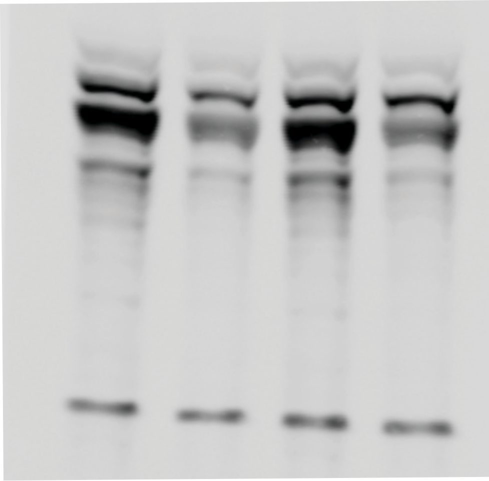

Gel stained with anti-GAPDH

1	2	3	4	5
Ladder	B16-F10 Cas9 WT	B16-F10 Cas9 MBNL1 KO	B16-F10 Cas9 MBNL2 KO	B16-F10 Cas9 MBNL DKO

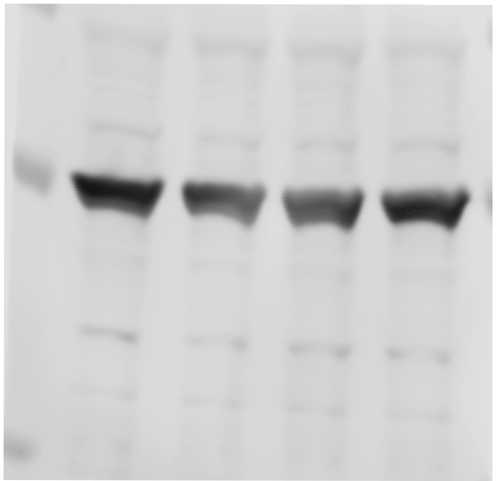

Gel stained with anti-MBNL2 antibody

1	2	3	4	5
Ladder	B16-F10 Cas9 WT	B16-F10 Cas9 MBNL1 KO	B16-F10 Cas9 MBNL2 KO	B16-F10 Cas9 MBNL DKO

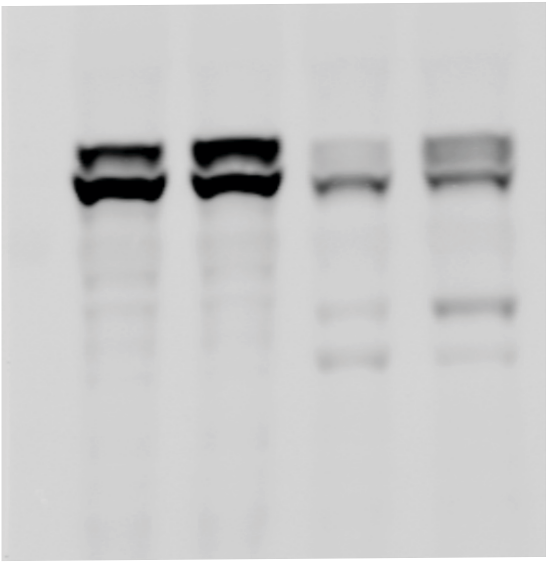

Supp. Figure 2, panel B

Red = GAPDH, Green = MBNL1

Lane	Sample
1	Ladder
2	B16-F10 Cas9 NTC Clone 1
3	B16-F10 Cas9 NTC Clone 2
4	B16-F10 Cas9 NTC Clone 3
5	B16-F10 Cas9 MBNL DKO Clone 1
6	B16-F10 Cas9 MBNL DKO Clone 2
7	B16-F10 Cas9 MBNL DKO Clone 3
8	B16-F10 Cas9 MBNL DKO Clone 4
9	B16-F10 Cas9 MBNL DKO Clone 5
10	B16-F10 Cas9 MBNL DKO Clone 6
11	B16-F10 Cas9 MBNL DKO Clone 7
12	Ladder

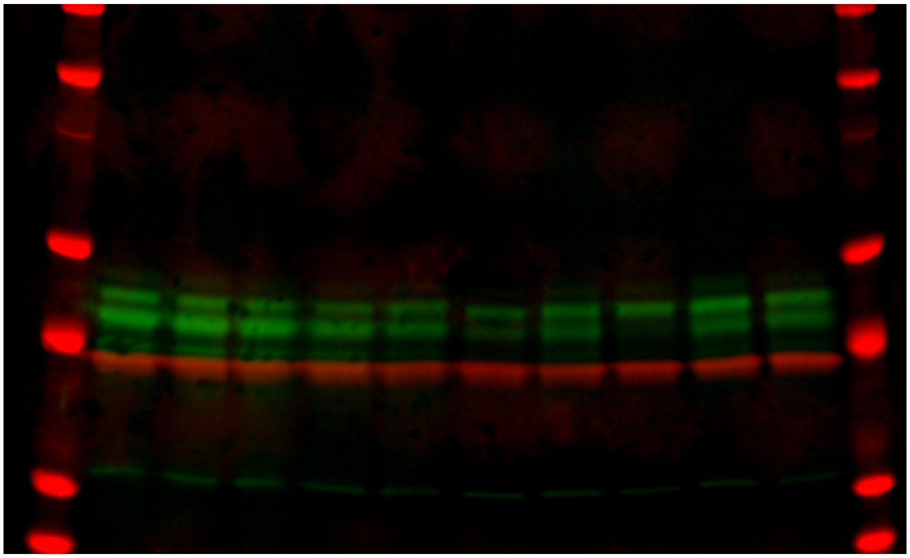

Red = GAPDH, Green = MBNL2

Lane	Sample
1	Ladder
2	B16-F10 Cas9 NTC Clone 1
3	B16-F10 Cas9 NTC Clone 2
4	B16-F10 Cas9 NTC Clone 3
5	B16-F10 Cas9 MBNL DKO Clone 1
6	B16-F10 Cas9 MBNL DKO Clone 2
7	B16-F10 Cas9 MBNL DKO Clone 3
8	B16-F10 Cas9 MBNL DKO Clone 4
9	B16-F10 Cas9 MBNL DKO Clone 5
10	B16-F10 Cas9 MBNL DKO Clone 6
11	B16-F10 Cas9 MBNL DKO Clone 7
12	Ladder

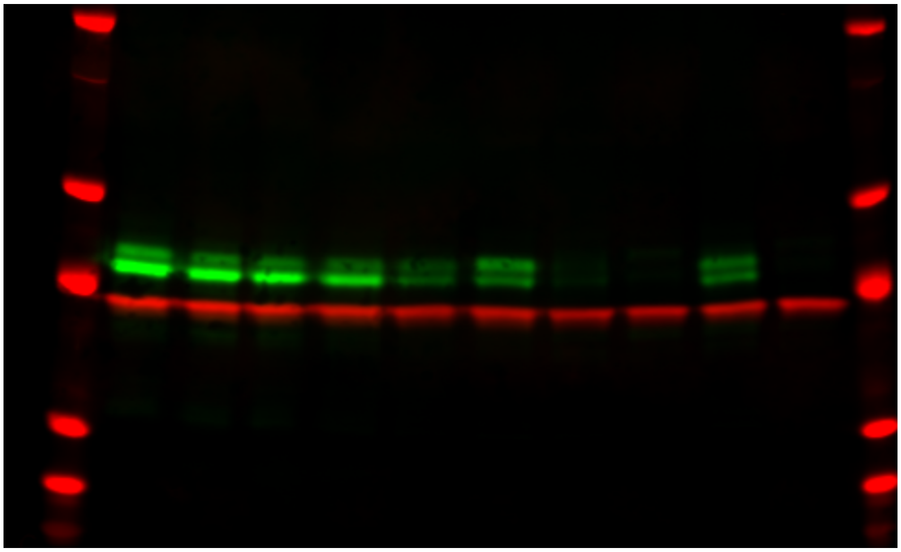

Supplement: S1 Raw Images — (PDF) [file pone.0321148.s002.pdf]
