# Supplementary material for: Muscleblind-like proteins are novel modulators of the tumor-immune microenvironment
Source: PLoS One. 2025 Apr 30;20(4):e0321148. doi: 10.1371/journal.pone.0321148 (PMC12043120; doi:10.1371/journal.pone.0321148)
Supplement: Attachment — Submitted filename: Review- PONE-D-24-15862.pdf [file pone.0321148.s006.pdf]

Dear Editor,

I have carefully reviewed the manuscript submitted to Plos One by Gabel AM and his colleagues. Their study entitled ‘Muscleblind-like proteins are novel modulators of the tumor-immune microenvironment’’ is that the effect of expression changes of a novel modulator, Muscleblind-like (MBNL) proteins, on the effect of CD8 T-cells on tumor cellsand tumor-immune microenvironment was investigated.
The study is well designed, many experiments have been carried out within the framework of the hypothesis and very valuable data have been obtained from the experiments. These have been systematically given in the results section of the article, but it is seen that the presented data have not been discussed in detail in the discussion section of the article in the light of the literature. Therefore, the valuable data obtained from the study should be detailly discussed in the section of the article. Also, there are a few minor grammatical errors in the article.

Best,
Best,
