# Supplementary material for: Muscleblind-like proteins are novel modulators of the tumor-immune microenvironment
Source: PLoS One. 2025 Apr 30;20(4):e0321148. doi: 10.1371/journal.pone.0321148 (PMC12043120; doi:10.1371/journal.pone.0321148)
Supplement: Attachment — Submitted filename: response_to_reviewers.pdf [file pone.0321148.s008.pdf]

Response to Reviews

Reviewer #1: I have carefully reviewed the manuscript submited to Plos One by Gabel AM and his colleagues. Their study entitled 'Muscleblind-like proteins are novel modulators of the tumor-immune microenvironment" is that the effect of expression changes of a novel modulator, Muscleblind-like (MBNL) proteins, on the effect of CD8 T-cells on tumor cells and tumor-immune microenvironment was investigated.

The study is well designed, many experiments have been carried out within the framework of the hypothesis and very valuable data have been obtained from the experiments. These have been systematically given in the results section of the article, but it is seen that the presented data have not been discussed in detail in the discussion part of the article in the light of the literature. Therefore, the valuable data obtained from the study should be detailly discussed in the section of the article. Also, there are a few minor grammatical errors.

Response:

We thank the reviewer for their careful review and kind comments regarding our manuscript. We appreciate their insights and have added significant detail to the discussion to put our findings more clearly into the context of the known role of MBNL proteins in regulating cancer and muscular dystrophy. We tie in the fact that most literature showing MBNL contributing to cancer phenotypes is related to regulation of alternative exon and intron inclusion or exclusion. We then review the known mechanisms of how splicing can alter signaling intensity of interferons. Lastly, we discuss evidence from previous studies linking MBNL dysregulation in muscular dystrophy to enhanced levels of inflammation. Addressing this comment has improved the quality of the manuscript, and we thank the reviewer for their suggestion.
